# Diagnosis and treatment of anterior urethral strictures in China: an internet-based survey

**DOI:** 10.1186/s12894-021-00950-0

**Published:** 2021-12-31

**Authors:** Changhao Hou, Yubo Gu, Wei Yuan, Zeyu Wang, Jiahao Lin, Qiang Fu, Lujie Song

**Affiliations:** 1 Department of Urology, Shanghai Jiao Tong University Affiliated Sixth People’s Hospital, Shanghai, 200233 China; 2 Shanghai Eastern Institute of Urologic Reconstruction, Shanghai, 200233 China

**Keywords:** Anterior urethral strictures, Practice pattern, Survey, Urethroplasty

## Abstract

**Background:**

To investigate the current diagnostic and therapeutic approaches to anterior urethral strictures of Chinese urologists and to compare with developed countries and the American Urologic Association guidelines.

**Methods:**

Anonymous questionnaires were distributed to members of Official Wechat Account of urology from March 19, 2020 to April 10, 2020. Descriptive and multiple correspondence analysis were used to analyze the data.

**Results:**

A total of 1276 online questionnaires were received. The response rate was 21.7% (1276/5878). The most common diagnostic methods for anterior urethral stricture were urethrography (90.7%) and urethrocystoscopy (85.4%), while urethral dilation (92.3%) and internal urethrotomy (60.1%) were the main therapeutic procedures. End-to-end urethroplasty (45.2%) was the most common open surgery, followed by skin flap urethroplasty (14.9%) and free graft urethroplasty (12.4%). 76.2% of urologists used urethroplasty only after the failure of minimally invasive surgery (reconstructive ladder treatment strategy). Furthermore, middle-aged or elderly urologists who had attended trainings, had senior practice roles, and who utilized a reconstructive ladder treatment approach were most likely to perform urethroplasties.

**Conclusions:**

Anterior urethral stricture treatment in China is still dominated by minimally invasive surgery, with most urologists using the reconstructive ladder treatment strategy. In general, the overall diagnostic and therapeutic strategies were similar between China and developed countries, with some deviations from the American Urologic Association guidelines.

**Supplementary Information:**

The online version contains supplementary material available at 10.1186/s12894-021-00950-0.

## Introduction

Although urethral strictures can be traced back to ancient times [1], here is still a poor understanding of this disease. According to recent data, 193–627 out of 100,000 men suffer from urethral strictures [2], with the morbidity and etiology of this disease varying greatly between different countries and regions [3, 4]. Trauma (51.67%), iatrogenic causes (34.49%), infection or inflammation (5.79%), and lichen sclerosus (4.22%) are the main etiologies of male urethral strictures [4]. Anterior urethral strictures are the most common, accounting for more than 90% of cases in the developed world [5, 6].

The clinical symptoms of patients with anterior urethral strictures are varied, however, the most apparent symptom is a weakening of the urinary stream. Although anterior urethral strictures can generally be diagnosed clinically, a stricture’s site, length, and scar thickness are more difficult to determine [7]. Therefore, urethroscopy, urethrography, urethral ultrasound, magnetic resonance imaging are used to characterize these lesions [7].

Urethral strictures can be treated surgically using two main approaches: minimally invasive surgery and urethroplasty. The former is generally favored by urologists because of its ease of application and simplicity. However, this approach has also been found to have an increasing number of complications and a high recurrence rate. Urethroplasty, on the other hand, has a high success rate and low recurrence rate; however, it has not been as widely studied [8]. The clinical management of urethral strictures remains controversial because of its diverse clinical manifestations, numerous surgical methods, various complications, and recent improvements in surgical instrumentation, clinician experience, and postoperative follow-up. Our previous study showed that the rates of endourological urethral surgery decreased significantly and the rates of urethroplasty increased during recent years.

The diagnostic and therapeutic approaches to anterior urethral strictures have been an ongoing focus of research. Investigators in the United States, Germany, Italy, and other developed countries have conducted related studies [9–13]. However, their findings do not necessarily reflect the situation in other regions. Furthermore, studies in developing countries are still lacking. As the largest developing country in the world, China has a vast territory, uneven economic development, and different levels of diagnosis and treatment in different regions. We conducted this study in order to better understand the diagnostic and therapeutic patterns of anterior urethral strictures in Chinese urologists and to explore the differences with developed countries and current guidelines.

## Methods

### Questionnaire

Based on prior performed surveys in the United States [10], the Netherlands [13] and in Italy [11], we modified a questionnaire suitable for Chinese urologists by referencing relevant literature and consulting experts in the field of urethral reconstruction. This survey queried respondents about demographic information, preoperative evaluations, surgery-related experience, and postoperative management using both single-choice and multiple-choice questions. Before the formal survey, we pilot-tested our survey on a sample of 60 urologists and finalized the 15-question survey (see Additional file 1) based on that feedback (e.g., eliminated outdated options, adjusting terminology).

Next, we use the largest Official Wechat Account of urology in China to distribute the survey. The members of this official account are all urologists. In order to ensure the efficiency of the survey, we set that each urologist can only response in once. One reminder was sent to all urologists. This study was conducted from March 19, 2020 to April 10, 2020 and approved by the ethics committee at our institution. All enrolled urologists signed a digital informed consent form before accessing the questionnaire online.

### Statistical analysis

The collected questionnaire data are presented as frequencies and percentages. Statistical analyses were performed using SPSS (version 20.0; SPSS Inc., Chicago, IL, USA). Multiple correspondence analysis (MCA) is a descriptive dimensionality reduction technique that employs categorical variables [14] (respondent demographics, surgery-related experience, and training). The results were explained according to parameters such as the total inertia. The total inertia represents the variability. The explanatory power of the variability provided ranges from 0 to 100% and the greater the variability, the greater the explanatory power. Statistical significance was defined as a *P* value of < 0.05.

## Results

### Respondent demographics

The questionnaire was distributed to 5878 urologists. A total of 1276 online questionnaires were received. The response rate was 21.7%. Excluding two incomplete questionnaires and seven respondents who lacked treatment experience with urethral strictures. In the end, 1267 valid questionnaires were obtained. Respondent age, practice role, hospital setting, etc. are shown in Table 1. The distribution of urologists in each province is shown in Additional file 1).

**Table 1:** Respondent demographics

Characteristics	No. of urologists	Percentage, %
*Age (yr)*		
< 30	70	5.5
30–39	538	42.5
40–49	484	38.2
50–59	164	12.9
≧60	11	0.9
*Practice role*		
Resident urologist	116	9.2
Attending urologist	471	37.2
Deputy chief urologist	451	35.6
Chief urologist	229	18.1
*Hospital setting*		
Grade A tertiary hospital	690	54.5
Grade B tertiary hospital	169	13.3
Secondary hospital	394	31.1
Inferior to secondary hospital	14	1.1
Hospital location*		
Northeast China	96	7.6
Central China	355	28.0
Eastern China	461	36.4
Western China	355	28.0
*Attended trainings*		
Yes	674	53.2
No	593	46.8

Hospital location*: *The northeast China* Heilongjiang Province, Jilin Province, Liaoning Province, Neimenggu Province. *The central China* Shanxi Province, Henan Province, Hubei Province, Hunan Province, Jiangxi Province, Anhui Province. *The eastern China* Beijing, Tianjin, Hebei Province, Shandong Province, Jiangsu Province, Shanghai, Zhejiang Province, Fujian Province, Guangdong Province, Hainan Province. *The western China* Chongqing, Sichuan Province, Guangxi Province, Guizhou Province, Yunnan Province, Shaanxi Province, Gansu Province, Ningxia Province, Xinjiang Province, Qinghai Province, Tibet Province

This survey involved urologists in all 31 provinces and municipalities of mainland China. The most common age group of respondents was between 30–39 years old. Attending urologists (31.2%) and associate chief urologists comprised the majority. A majority (54.5%) of urologists worked in grade A tertiary hospitals, while 408 urologists worked in secondary hospitals or those inferior to secondary hospitals. Of all respondents, 53.2% of urologists stated that they had participated in training related to urethral repair and reconstruction.

### Preoperative evaluation and surgery related experience

Of all respondents, 52% believed that the maximum length of stricture for internal urethrotomy (IU) was 1 cm or less, while 277 believed that < 2 cm was appropriate, and 25 believed that < 3 cm was appropriate. In addition, 7.9% of urologists stated that stricture length was not the main criterion for IU. Among the types of IU, 57.1% of urologists tended to use cold knife for IU and 20.8% tended to use laser IU. When performing intraoral mucosal urethroplasty, more than half of urologists (687/1267, 54.2%) preferred the buccal mucosa, followed by the lingual mucosa (430/1267, 33.9%), lower lip mucosa (123/1267, 9.8%), and upper lip mucosa (27/1267, 2.1%). When reporting their greatest concern while performing urethroplasty, most urologists were concerned about postoperative complications, such as sexual function and postoperative infection, with the minority of urologists reporting the difficulty of the surgery. Preoperative evaluation and surgery related experience are shown in Table 2.

**Table 2:** Approach to preoperative evaluations for anterior urethral strictures and surgery-related experience of Chinese urologists

		No. of urologists	Percentage, %
Treatment strategy	Reconstructive ladder treatment strategy^a^	965	76.2
	Performance of a primary Urethroplasty^b^ if indicated	302	23.8
What methods do you usually use to diagnose anterior urethral strictures? (multiple choice)	Uroflowmetry	680	53.7
	Postvoid residual urine	548	43.3
	Urethroscopy	1076	84.9
	Urethrography	1143	90.2
	Urethral ultrasound	127	10.0
	Trial catheterization	699	55.2
	Trial urethral dilation	762	60.1
	Other	23	1.8
Which of the following procedures have you performed in the last year? (multiple-choice)	Urethral dilation	1170	92.3
	Internal urethrotomy	762	60.1
	Urethral anastomosis	573	45.2
	Skin-flap urethroplasty	189	14.9
	Free-graft urethroplasty	157	12.4
	Endourethral stent	136	10.7
	Perineal urethrostomy	142	11.2
	Urethral realignment	548	43.3
	Urethral meatotomy	427	33.7
	Other	11	0.9
No. of urethroplasties	0	384	30.3
	1–5	595	47.0
	6–10	175	13.8
	11–20	53	4.2
	> 20	60	4.7
Greatest concern when performing urethroplasty	Major hemorrhage during the operation	187	14.8
	Difficulty obtaining samples from the oral mucosa	281	22.2
	Trauma during oral mucous membrane sampling	135	10.7
	Difficulty obtaining genital skin flap from an	260	20.5
	unclear dissection	614	48.5
	Influencing sexual function	306	24.2
	Postoperative infection	632	49.9
	Urethral restenosis	1167	92.1
	Other	36	2.8

^*a*^*Reconstructive ladder strategy* Starting with minimally invasive procedures (including dilatation/urethrotomy) and only considering open urethroplasty after failure of the initial approach

^*b*^*Urethroplasty* Urethral anastomosis, skin-flap urethroplasty, free-graft urethroplasty, urethral traction, perineal urethrostomy, urethral realignment, urethral meatotomy

### Postoperative management

After IU, catheters were retained for 2 weeks by 40.1% of urologists, 72 h by 2.6%, 1 week by 12.1%, 3 weeks by 9.7%, 4 weeks by 27.5%, and more than 4 weeks by 9.9%. After urethral reconstruction, 32.6% of urologists indwelled catheters for 2 weeks, 15% indwelled for 3 weeks, 32% indwelled for 4 weeks, and 13% indwelled for more than 4 weeks, with 5% removing the catheter after 1 week. Six urologists believed that catheters could be removed after 72 h.

### Correspondence of respondent demographics, surgery related experience and training

Based on the above survey results, we identified variables with statistical differences, including age (*P* < 0.01), practice role (*P* < 0.01), hospital location (*P* < 0.01), treatment strategy (*P* < 0.01), management (*P* < 0.01) and number of urethroplasties (*P* < 0.01). Hospital setting (*P* = 0.129) was excluded. We then used the MCA technique to show correlations between categorical variables. MCA is a descriptive dimensionality reduction technique that employs categorical variables. The positions of the categories of each variable in the multidimensional plane can be used to determine groups with similar patterns through graphical representation [15]. Then, a hierarchical cluster analysis was performed from the coordinates obtained in the MCA to confirm the verified groups by proximity in the visual inspection. Next, a hierarchical cluster analysis was performed from the coordinates obtained in the MCA map according to the proximity in the visual inspection [16]. The closer the distance between the two variables is, the stronger the correlation between the two variables is.

**Fig. 1:**
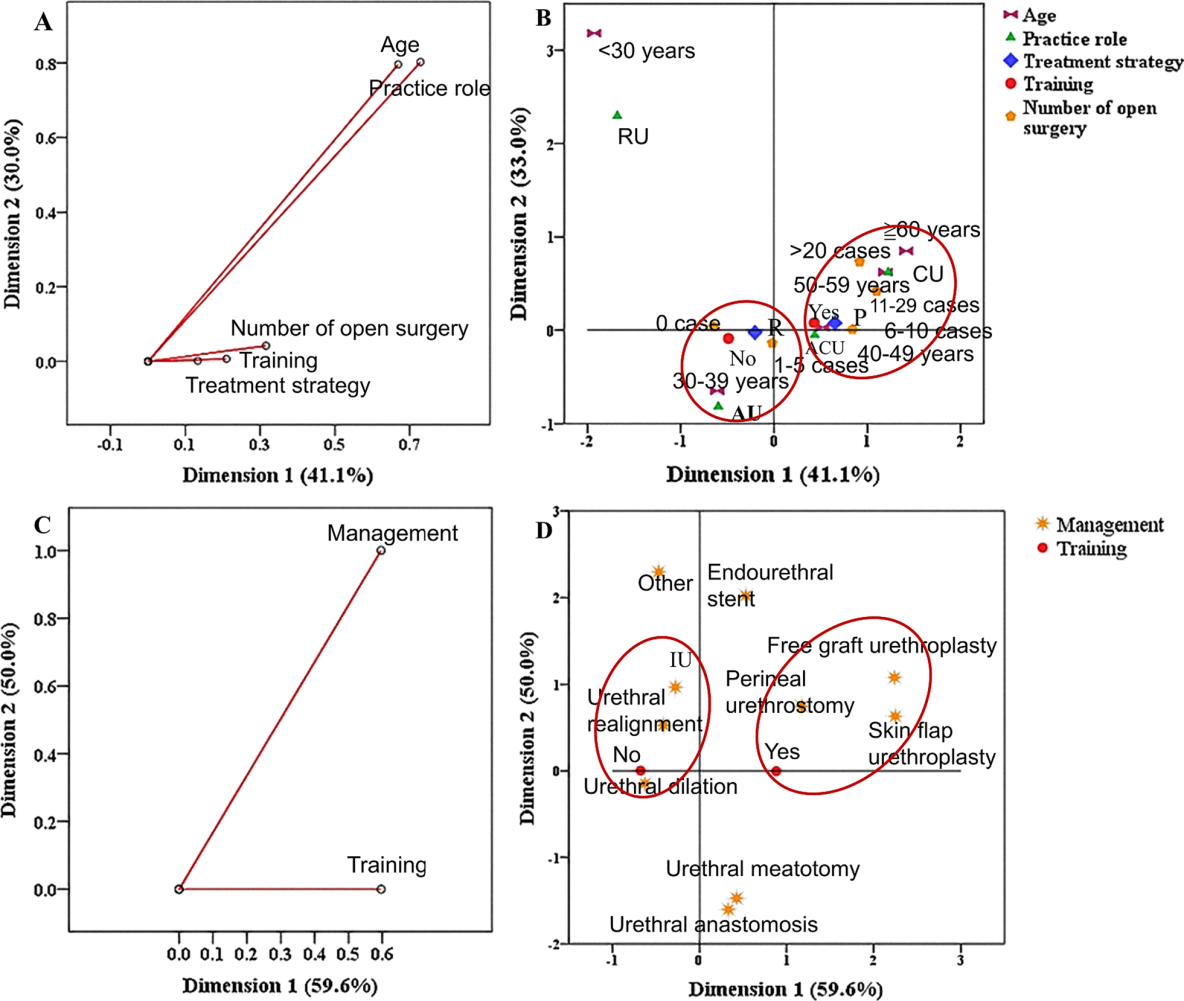
Correspondence of respondent demographics, surgery related experience and training. **A** Discrimination measures graph: training and respondent demographics. **B** MCA map between training and respondent demographics. **C** Discrimination measures graph: training and management. **D** MCA map between training and management. MCA was used to explore the correspondence of respondent demographics, surgery related experience and training. These figures show the correlations between the scattered points of each variable in each category. The closer the distance between the scattered points, the more obvious the correlation. (*RU* resident urologist, *CU* chief urologist, *AU* attending urologist)

In the MCA of respondent demographics and training, the inertia for all variables in the two dimensions were 0.300 and 0.411, respectively (Fig. 1A). The total inertia corresponding to the two dimensions in the MCA explained for 71.1% of the total data variability. The MCA map (Fig. 1B) for urologists who did not attend training showed that the following characteristics were associated with this group: performed 0 or 1–5 cases, 30–39 years of age, attending urologist, and the reconstructive ladder treatment strategy. In contrast, the group of urologists who had attended training were associated with the following characteristics: performed 6–10, 11–20, or ≧20 cases; 40–49, 50–59, or ≧60 years of age; associate chief or chief urologist; and a non-reconstructive ladder treatment strategy.

In the MCA of surgery-related experience and training, the inertia for all variables in the two dimensions were 0.596 and 0.500, respectively (Fig. 1C). Urethral dilation, urethral realignment, and IU were associated with urologists who had not attended training, while perineal urethrostomy, skin flap urethroplasty, and free graft urethroplasty were associated with urologists who had attended training (Fig. 1D). There was a strong correlation between these factors, demonstrating that urologists who had attended training were significantly more likely to perform open surgeries.

## Discussion

To the best of our knowledge, this survey included the largest number of participants of any existing survey related to anterior urethral strictures and was the first survey of anterior urethral strictures conducted in China, Asia, or any developing country. It was also the first internet-based anterior urethral stricture survey. Internet-based surveys can overcome the limitations of traditional questionnaires, allowing for the collection of more samples at a lower cost over less time. It is worth mentioning that the urologists who participated in our survey were not limited to specialists in the field of urethral repair and reconstruction, since, in China, both general urologists and specialists participate in the diagnosis and treatment of this disease. Therefore, our study better reflects the contemporary characteristics of the diagnosis and treatment of anterior urethral strictures in China.

**Fig. 2:**
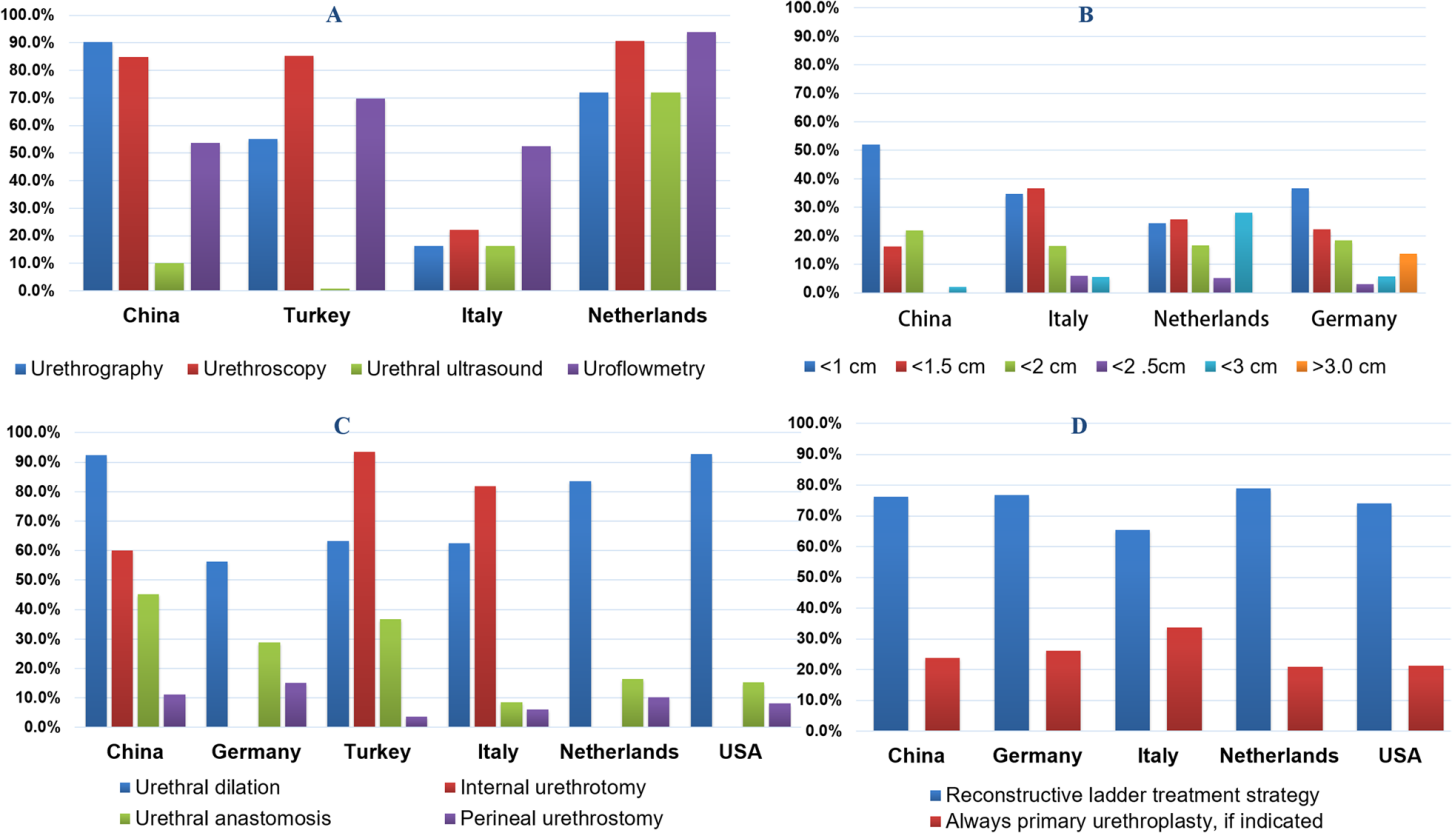
Comparison of diagnostic and therapeutic approaches to anterior urethral strictures in different countries. **A** Comparison of diagnostic methods for anterior urethral strictures in different countries. **B** Maximum stricture lengths for IU in different countries. **C** Surgical procedures performed for anterior urethral strictures in the last year; and **D** treatment strategies in different countries

Although it has been 13 years since the first related survey in the USA [10], our results found that the overall diagnostic and therapeutic approaches to anterior urethral strictures in China have not changed much in comparison to developed countries [9–13]. There were, however, some discrepancies with American Urologic Association (AUA) guidelines [17]. In terms of the preoperative diagnosis, 78% of Chinese urologists did not rely on a single diagnostic procedure but rather used multiple methods, which is consistent with the AUA guidelines that one individual test cannot make a definitive diagnosis and that retrograde urethrography should be the primary choice [17]. Urethrography was the most common diagnostic method (90.7%) reported in this survey, which was higher than has been reported in other countries, including the Netherlands (72%), Turkey (55%), and Italy (22%). The percentage of urologists utilizing urethroscopy was similar to what has been reported in prior studies. Compared with the Netherlands (93.8%) and Turkey (69.7%), the utilization rate of uroflowmetry (53.7%) was low (Fig. 2A), perhaps because, in patients with typical symptoms of an anterior urethral stricture (e.g., dysuria) some urologists may not think a uroflowmetry examination is necessary. Additionally, 10.1% of urologists used urethral ultrasonography. Furthermore, a considerable number of urologists adopted invasive approaches during evaluations, such as trail urethral dilation and trail catheterization, which has not been mentioned in previous surveys or AUA guidelines.

Increasing evidence demonstrates that urethroplasty is the gold standard for treatment of urethral strictures [18]. However, our survey results showed that urethral dilation (92.3%) and IU (60.1%) were still the most commonly used procedures by Chinese urologists, which is consistent with all previous surveys. While Chinese urologists most commonly used urethral dilation, previous studies have shown a tendency to use IU (Germany, 87.2%; Turkey, 93.5%; China, 60.1%) (Fig. 2C). More than half of urologists preferred to use cold-knife IU, which is in accordance with our clinical practice. Also, only about one-fifth of respondents followed the AUA guideline [17] that the maximum suitable length for IU is less than 2 cm (Fig. 2B), which was higher than in developed countries, such as the Netherlands (16.6%), Germany (18.4%), and Italy (16.4%). AUA guidelines also recommend removing the urethral catheter within 72 h following a dilation or direct visual internal urethrotomy [17]. However, only 2.6% of our respondents followed this recommendation.

An article about learning curves of urologists pointed out that the average number of anterior urethroplasties required to reach proficiency (defined as a success rate of > 90%) is approximately 100 cases [19]. To our surprise, 33.3% of respondents stated that they had not performed urethroplasties, compared with 77% in the Netherlands, 73.2% in Germany, 60.8% in Italy, and 57.8% in the USA. However, the majority of respondents (77.3%) had performed less than five urethroplasties in the prior year, with only eight (8.9%) performing more than 10 cases. Therefore, most respondents were likely unfamiliar with urethroplasty. The rate of urethroplasty may also have been low because of financial factors, with some urologists believing that the cost of IU is less than that of urethroplasty. Although our survey did not address this problem, existing models have proven that urethroplasty is economically advantageous [19]. Santucci [20] stated that, if urethroplasty is not performed, patients with urethral stricture should be referred to urologists with expertise, instead of being treated with futile repeated dilation, which is similar to the expert opinions in the AUA guidelines. Furthermore, about two-thirds of urologists have performed urethroplastyies, but some still prefer minimally invasive surgery, such as urethral dilatation or IU. In fact, this is consistent with the treatment strategy of anterior urethral stricture that we learn from our survey.

In our survey, 72.2% of urologists considered urethroplasty only after failure of minimally invasive surgical approaches, with 23.8% believing that urethroplasty should be performed first if the indication is appropriate, which was almost identical to the rates in Germany, Italy, the Netherlands, and the USA (Fig. 2D). In addition, the practice role, hospital location, and participation in training were significantly different based on the treatment strategy (*P* < 0.01). Bullock et al. [10] considered that the “reconstructive ladder” strategy is erroneous, educating urologists to understand the high reference rate of successive IU and the myth of the reconstructive ladder is critical. But Clemens [12] argued that this treatment strategy is justifiable and the most cost-effective approach for short bulbar strictures. Topakta et al. [21] analyzed 133 patients who underwent urethroplasty for bulbar urethral strictures and evaluated the effects of a previous IU, demonstrating that the success rates for urethroplasties could be affected by a previous history of any endoscopic procedures, which suggests that a non-reconstructive ladder treatment approach may be more appropriate.

The MCA technique is a multivariate statistical analysis that can describe the correlation between different variables or between different categories of the same variable [15]. An MCA map can intuitively reflect the relationship between variables, especially for categorical variables. Based on the MCA results, there were significant differences in respondent demographics and surgery-related experience between urologists who had attended training and those who had not. Urologists were more likely to perform urethroplasties if they had attended training were in senior practice roles, were middle-aged or elderly, or if they utilized the reconstructive ladder treatment approach. Michael et al*.* [22] conducted an anonymous survey about barriers to accessing urethroplasty, with the results suggesting that the best predictor of a urethroplasty recommendation was formal training in urethroplasty. Therefore, we speculate that strengthening training may help to improve the diagnostic and therapeutic level to anterior urethral strictures. As the largest urethral repair referral center in China, our institution plays an important role in this task.

It should, however, be noted that our study has some limitations. First, our survey does not cover all the questions about the diagnosis and treatment of anterior urethral stricture, because we have to consider the answer time of the questionnaire. Second, the content chosen by urologists represents their main views on the diagnosis and treatment of anterior urethral stricture, but many factors in clinical practice may affect their final decision. Therefore, there may be differences in the actual treatment measures adopted by urologists and the choice of questionnaires. In addition, our response rate was low (21.7%). It is worth mentioning that the urologists who participated in our survey were general urologists. A certain percentage of urologists are not familiar with the field of urethral repair and reconstruction. This is the main reason why the response rate is not high. Our survey cannot guarantee that the respondents who participated in this survey were representative of general urologists in China in terms of age, practice role, hospital setting, etc., which may be related to our use of online surveys.

## Conclusions

In China, anterior urethral strictures are primarily managed by minimally invasive surgery and the majority of urologists use the reconstructive ladder treatment strategy. The overall diagnostic and therapeutic strategies for anterior urethral strictures were similar between China and developed countries, with some deviations from the AUA guidelines. The standard of care should be in line with guidelines in managing urethral stricture disease. Finally, strengthening relevant training for urologists may help to improve the diagnostic and therapeutic approaches to anterior urethral strictures.

## Supplementary Information

**Additional file 1.** The questionnaire and the distribution of urologists in each province.

## Data Availability

The data sets used and/or analyzed during the current study are available from the corresponding author on reasonable request.

## Abbreviations

AUA: American Urologic Association
IU: Internal urethrotomy
MCA: Multiple correspondence analysis
RUG: Retrograde urethrography
