# Supplementary material for: Diagnosis and treatment of anterior urethral strictures in China: an internet-based survey
Source: BMC Urol. 2021 Dec 31;21:185. doi: 10.1186/s12894-021-00950-0 (PMC8720216; doi:10.1186/s12894-021-00950-0)
Supplement: Supplementary file 1 — Additional file 1. The questionnaire and the distribution of urologists in each province. [file 12894_2021_950_MOESM1_ESM.docx]

**Supplementary**

**Questionnaire on diagnosis and treatment of anterior urethral stricture in China**

1. **Your age group：**

• <30 years

• 30-39 years

• 40-49 years

• 50-59 years

• ≧60 years

1. **Your practice role:**

• Resident urologist

• Attending urologist

• Associate chief urologists

• Chief urologist

1. **Your hospital setting:**

• Grade-A tertiary hospital

• Grade-B tertiary hospital

• Secondary hospital

• Below secondary hospital

1. **Your hospital location (Provinces and cities):___________**

1. **Have you ever attended training course related to urethral repair and reconstruction?**

• Yes

• No

1. **Which treatment strategy do you think is better for patients with anterior urethral stricture?**

• Starting with minimally invasive procedures (including dilatation/urethrotomy), only considering open urethroplasty after failures.

• Always primary urethroplasty, if indicated

1. **What methods do you usually use to dignose anterior urethral sticture?**

• Uroflowmetry

• Postvoid residual urine

• Urethrocystoscopy

• Urethrography

• Urethral ultrasound

• Trial catheterization

• Trial urethral dilation

• Other (Please describe):**___________**

1. **Which of the following procedures have you performed in the last year?**

• Urethral dilation

• Internal urethrotomy

• Urethral anastomosis

• Skin flap urethroplasty (Such as genital flap)

• Free graft urethroplasty (Such as tongue mucous, lip mucosa, buccal mucosa, etc.)

• Endourethral stent

• Perineal urethrostomy

• Urethral realignment

• Urethral meatotomy.

• Other (Please describe):**___________**

1. **Number of open urethroplasties you have performed in the last year?**

• 0

• 1–5

• 6–10

• 11–20

• >20

1. **What is your greatest concern when you performed open urethrolplasty in anterior urethral stricture?**

• Major hemorrhage during operation

• It is difficult to obtain samples from oral mucosa

• The trauma of oral mucous membrane sampling is great

• It is difficult to obtain genitals skin flap from

• It is not clear to dissect the narrow part of

• Influencing sexual function

• Postoperative infection of

• Urethral restenosis

• Other (Please describe):**___________**

1. **What do you think is the maximum suitable stricture length for internal urethrotomy?**

• <1 cm

• <1.5 cm

• <2 cm

• <3 cm

• The stricture length is not the main criterion for choosing internal urethrotomy

1. **What do you think is the best types for internal urethrotomy ?**

• Cold knife

• Laser

• Electric cutting

• Injection of anti-scarring drugs after incision

• Other(Please describe):**___________**

1. **How long do you think the catheter should be retained after internal urethrotomy ?**

• 72 hours

• 1 week

• 2 weeks

• 3 weeks

• 4 weeks

• More than 4 weeks

1. **How long do you think the catheter should be retained after open urethrolplasty ?**

• 72 hours

• 1 week

• 2 weeks

• 3 weeks

• 4 weeks

• More than 4 weeks

1. **When performing intraoral mucosal urethroplasty, where do you prefer to choose the material or what do you think is more reasonable ?**

• Buccal

• Tongue

• Lower lip

• Upper lip

| **Table R1** The distribution of urologists in each province | |
| --- | --- |
| **Province** | No. of urologists |
| Anhui | 46 |
| Beijing | 26 |
| Fujian | 25 |
| Gansu | 26 |
| Guangdong | 74 |
| Guangxi | 26 |
| Guizhou | 37 |
| Hainan | 5 |
| Hebei | 64 |
| Henan | 91 |
| Heilongjiang | 36 |
| Hubei | 51 |
| Hunan | 87 |
| Jilin | 22 |
| Jiangsu | 47 |
| Jiangxi | 29 |
| Liaoning | 19 |
| Neimenggu | 19 |
| Ningxia | 8 |
| Qinghai | 9 |
| Shandong | 92 |
| Shanxi | 51 |
| Shaanxi | 50 |
| Shanghai | 62 |
| Sichuan | 85 |
| Tianjin | 7 |
| Xizang | 1 |
| Xinjiang | 28 |
| Yunnan | 66 |
| Zhejiang | 59 |
| Chongqing | 19 |
